# Assessment of clinically relevant drugs as feline P-glycoprotein substrates

**DOI:** 10.3389/fvets.2025.1668282

**Published:** 2025-10-08

**Authors:** Katrina L. Mealey, Neal S. Burke

**Affiliations:** Program in Individualized Medicine (PrIMe), Department of Veterinary Clinical Sciences, Washington State University, Pullman, WA, United States

**Keywords:** P-glycoprotein, MDR1, eprinomectin, cisapride, feline

## Abstract

**Introduction:**

The drug transporter P-glycoprotein (P-gp) influences drug disposition by playing key roles in limiting brain penetration and enhancing biliary excretion of substrate drugs. Guidance documents from U.S. and European regulatory agencies recommend that manufacturers determine the P-gp substrate status of new medications intended for human patients. The rationale is that P-glycoprotein-mediated drug–drug interactions may cause serious adverse drug events. Unfortunately, the same regulatory guidance does not encompass new feline drugs even though a P-gp knockout mutation (ABCB11930_1931del TC) is present in a subpopulation of cats. Recent reports of a novel macrocyclic lactone, eprinomectin, causing neurological toxicosis in cats homozygous for ABCB11930_1931del TC, imply it is a feline P-gp substrate, but definitive data is lacking, It is intriguing that neurological toxicity has also been reported in a small number of cats treated with amlodipine, capromorelin, and cisapride, however their MDR1 genotypes are unknown.

**Methods:**

A competitive efflux assay and feline P-gp expressing cell line were used to assess the P-gp substrate status of thirteen clinically important drugs used in cats.

**Results:**

Ten drugs, including eprinomectin, were determined to be substrates for feline P-gp while three drugs were not.

**Discussion:**

This information will help improve drug safety for cats with intrinsic (ABCB11930_1931del TC) and acquired P-gp deficiency. Further, this type of assay may be useful for screening feline drug candidates during the drug approval process.

## 1 Introduction

P-gp, the ABCB1 (MDR1) gene product, markedly influences the disposition of dozens of drugs in mammals (1, 2). P-gp is expressed on many feline tissues including the intestines, biliary canalicular cells, and brain capillary endothelial cells where it functions to actively extrude substrate drugs (3). Consequently, P-gp protects cats and other mammals from substrate drugs by enhancing their biliary excretion and restricting their entry into the central nervous system (4, 5) thereby preventing many serious adverse drug reactions. A P-gp-null mutation was discovered in dogs in 2001 (ABCB1-1Δ) (6) and a different P-gp-null mutation was discovered in cats in 2015 (*ABCB1*1930_1931del TC) (7). Because the canine MDR1 mutation was identified fourteen years before the feline MDR1 mutation was discovered, there is more information regarding adverse drug reactions caused by P-gp substrate drugs in dogs with the canine MDR1 mutation, including parasiticides, anticancer drugs, gastrointestinal drugs, immunosuppressants, sedatives and others (reviewed in 2). Whether cats with P-gp deficiency are susceptible to those same drugs is not known. Species differences in the amino acid sequence of P-gp’s substrate binding pocket causes species differences in the P-gp substrate status of various drugs (8, 9). While there is substantial overlap, drugs that are canine P-gp substrates may not be human, murine, or feline P-gp substrates. Therefore, prevention of serious adverse drug reactions in cats with P-gp deficiency can only be accomplished by knowing which drugs are substrates for feline P-gp.

A case in point is represented by the serious adverse drug reactions caused by the macrocyclic lactone, eprinomectin, included in several relatively new feline parasiticide products^1^^,^^2^^,^^3^. These products, formulated specifically for cats have caused serious, even fatal, adverse neurological reactions in P-gp deficient cats when used in accordance with label instructions (10, 11). One would expect eprinomectin to be a substrate for feline P-gp because other macrocyclic lactones have been documented to be substrates for canine, human, and murine P-gp. However, the status of eprinomectin as a feline P-gp substrate has not actually been assessed.

The central hypothesis provoking this study was that many drugs used in veterinary practice are feline P-gp substrates and that use of these drugs in P-gp deficient cats may cause serious, but preventable, adverse drug reactions. The aim of the study reported here was to initiate development of an efficient *in vitro* screening assay to determine the feline P-gp substrate status of thirteen clinically important feline drugs (Table 1).

**Table 1:** Drugs screened as feline P-gp substrates and rationale for their inclusion.

Drug	Rationale
Amlodipine	Human P-gp substrate (26); reports of sporadic CNS toxicity when prescribed to cats (27).
Capromorelin*	Structure suggestive of P-gp substrate; 13% of cats treated with capromorelin developed lethargy and 17% developed behavior changes^a^.
Cefovecin*	Potential cause of P-gp-mediated drug–drug interaction in a cat (10); other cephalosporins are P-gp substrates in other species (28).
Cisapride	P-gp substrate in other species (29); sporadic reports of neurological toxicity when prescribed to cats^b^.
Cisplatin	Negative control.
Cyclosporine*	Potential cause of P-gp-mediated drug–drug interaction in a cat (10); Dogs with the MDR1 mutation are susceptible to adverse effects (30).
Doxycycline	Potential cause of P-gp-mediated drug–drug interaction in a cat (10); other tetracyclines are P-gp substrates in other species (31).
Emodepside*	Causes neurologic toxicity in dogs and mice with P-gp deficiency (32, 33)
Eprinomectin*	Causes neurological toxicity in cats with P-gp dysfunction (10, 11).
Ivermectin*	Positive control – Causes neurological toxicity in cats with P-gp dysfunction (7, 34).
Loperamide	Positive control; causes severe CNS depression in dogs with P-gp deficiency (5).
Methylprednisolone*	Potential cause of P-gp-mediated drug–drug interaction in a cat (10); P-gp substrates in other species (35).
Ronidazole	Structure suggestive of P-gp substrate; sporadic reports of CNS toxicity in cats (36).
Selamectin*	P-gp substrate in other species (37).
Vinblastine	Causes severe myelosuppression and GI toxicity in P-gp deficient dogs (38).
Vincristine	Causes severe myelosuppression and GI toxicity in P-gp deficient dogs (39).

a https://www.elancolabels.com/us/elura (accessed April 15, 2024).

b https://cats.com/cisapride-for-cats (accessed April 23, 2024). *Drug is available in an FDA and/or EMA approved product labeled for cats.

## 2 Materials and methods

### 2.1 Cell lines and culture conditions, drugs, and chemicals

Crandell-Rees Feline Kidney (CRFK) and HeLa cells were obtained from American Type Culture Collection (Manassas, VA); hMDR1 P-gp cells, in which canine P-gp is knocked out and human P-gp is overexpressed, were a generous gift from the Karlgren laboratory (Upsala, Sweden) (12); MDCK PGP cells, Madin Darby Canine Kidney cells which were previously induced to overexpress canine P-gp were generated by our laboratory (12). Cells were maintained in Dulbecco’s modified eagle medium (DMEM) containing 10% fetal bovine serum. Doxorubicin hydrochloride, rhodamine 123, calcein AM, amlodipine, cisapride, cisplatin, cyclosporine A, emodepside, eprinomectin, ivermectin, MK-571, methylprednisone, ronidazole, selamectin, vinblastine, and vincristine were purchased from Tocris Biosciences (Minneapolis, MN). The fluorescent MRP1 substrate carboxyfluorescein diacetate (CFDA) was purchased from Invitrogen (Carlsbad, CA).

### 2.2 Western blot assessment of P-gp and multidrug resistance-associated protein 1 (MRP1) expression in CRFK cells

Total protein concentrations for hMDR1MDCK, CRFK, and HeLa whole cell lysates were determined using Pierce BCA protein assay kit (Thermo Scientific, Rockford, IL). Twenty micrograms of total protein from each cell lysate were separated on 4–15% Tris–HCl gradient polyacrylamide gels (Invitrogen, Carlsbad, CA) and electrotransferred to polyvinylidene difluoride membranes. The membranes were incubated in blocking buffer [0.5% fish gelatin in tris buffered saline (TBS)] for 1 h at room temperature, then incubated with primary antibody (1:40 C219 for P-gp; Invitrogen, Carlsbad, CA or 1:100 ab3368 for MRP; Abcam, Cambridge, MA) for detection of P-gp or MRP1, respectively, overnight at 4 C. The membrane was washed in blocking buffer with 0.05% Tween 20 and incubated with horse radish peroxidase-conjugated secondary antibody (Thermo Pierce, Rockford, IL) for 60 min at 4 °C. After a final wash, secondary antibody binding was detected using Immobilon Western Chemiluminescent HRP substrate (Millipore, Burlington, MA).

### 2.3 Confirmation of functional P-gp in CRKF cells using rhodamine 123 efflux competition assay

The well-characterized human P-gp-expressing cell line hMDR1-MDCK (13) was used as a standard to compare with feline P-gp function in CRFK cells. hMDR1 MDCK and CRFK cells were plated and incubated overnight in 12-well plates. Media containing either vehicle alone, positive control P-gp substrate (loperamide or ivermectin), the negative control non-P-gp substrate (cisplatin), the fluorescent P-gp substrate rhodamine 123 alone, ivermectin + rhodamine 123, loperamide + rhodamine 123 or cisplatin + rhodamine 123 were added to individual wells. Plates were incubated for 1 h under standard cell culture conditions (described above). After incubation, media was aspirated off the cells, the cells were trypsinized and washed with ice cold phosphate buffered saline (PBS). Cells were pelleted by gentle centrifugation and the pellet was resuspended in 200 μL PBS. Cells were then subjected to flow cytometry. Cells were analyzed on a Guava easyCyte flow cytometer (Cytek Biosciences, Fremont, CA) with an argon laser set at 488 nm. A minimum of 10,000 events were collected for each well. The fluorescence emission of rhodamine 123 was collected in the FL1 channel with 530/30 bandpass filter. Triplicate wells of each drug/combination were analyzed, and all experiments were repeated on at least one additional, separate day to ensure repeatability.

### 2.4 Competitive efflux assay with fluorescent P-gp substrates rhodamine 123 and calcein AM

Table 1 lists the positive and negative controls, experimental drugs and the three different concentrations of each drug used in these experiments. The intent was to incorporate a therapeutic or Cmax plasma concentration, if known, for experimental drugs, and at least two higher concentrations. Prior to efflux assays, drugs to be used for the day were dissolved in vehicle (PBS for cisplatin, and vincristine; ethanol for loperamide and PSC 833; dimethylsolfoxide [DMSO] for all other drugs) and diluted to 1,000X the highest concentration to be tested. For assays, stock solutions were diluted serially 1:10 in media to yield the indicated experimental concentrations. The concentration of vehicle in controls and fluorescent probe only wells was normalized to that of the vehicle concentration at the highest concentration of experimental drug. Except for selamectin, the maximum concentration of vehicle in each well was 0.1%. For selamectin, the concentration of DMSO was 2%.

CRFK cells were plated and incubated overnight in 12-well plates. For efflux competition assays rhodamine 123 was studied first for each experimental drug. If the drug did not compete with rhodamine 123 (an R-site P-gp substrate) for P-gp mediated efflux, then the fluorescent P-site P-gp substrate calcein AM was used. Media containing the fluorescent P-gp substrate (10 μM rhodamine 123 or 1 μM calcein AM) alone or the fluorescent substrate plus the indicated concentration (Table 1) of each control or experimental drug were added to wells. The competitive efflux assay was performed as described in the previous section. Triplicate wells of each drug/combination were analyzed and all experiments were repeated on an additional, separate day to ensure repeatability. The mean fluorescence intensity (MFI) of cells from each well was used to determine the MFI ratio, which is the ratio of fluorescence intensity in cells treated with fluorescent P-gp substrate plus experimental drug to that of cells treated with the fluorescent P-gp substrate only [MFI (experimental drug + rhodamine): MFI (rhodamine alone)]. The highest MFI ratio achieved for an experimental drug was used to assess its P-gp status. Drugs were considered non P-gp substrates if the MFI ratio was < 2; weak P-gp substrates if the MFI was ≥ 2 but < 5: moderate P-gp substrates if the MFI was > 5 but less than 10; and strong P-gp substrate if the MFI was ≥ 10.

### 2.5 Assessment of calcein AM competitors as MRP1 substrates

For drugs that competed with calcein AM for P-gp-mediated efflux, additional efflux studies were carried out to determine if those drugs were MRP1 substrates using the selective, fluorescent MRP1 substrate CFDA as previously described (14). Briefly, plated cells were incubated with either CFDA (10 μM) alone or CFDA plus the specific MRP inhibitor MK-571 (100 μM) (15), CFDA plus amlodipine (2.5 μM) or CFDA plus methylprednisolone (100 μM). Plates were incubated for 1 h under standard cell culture conditions (described above). After incubation, the media was aspirated off and cells were trypsinized and washed with ice cold phosphate-buffered saline (PBS). Cells were pelleted by gentle centrifugation and the pellet was resuspended in 200 μL PBS. Cells were then subjected to FACS as described above. The MFI of cells from each well was used to determine the MFI ratio.

## 3 Results

### 3.1 Western blot assessment of P-gp and MRP1 expression in CRFK cells

**Figure 1:**
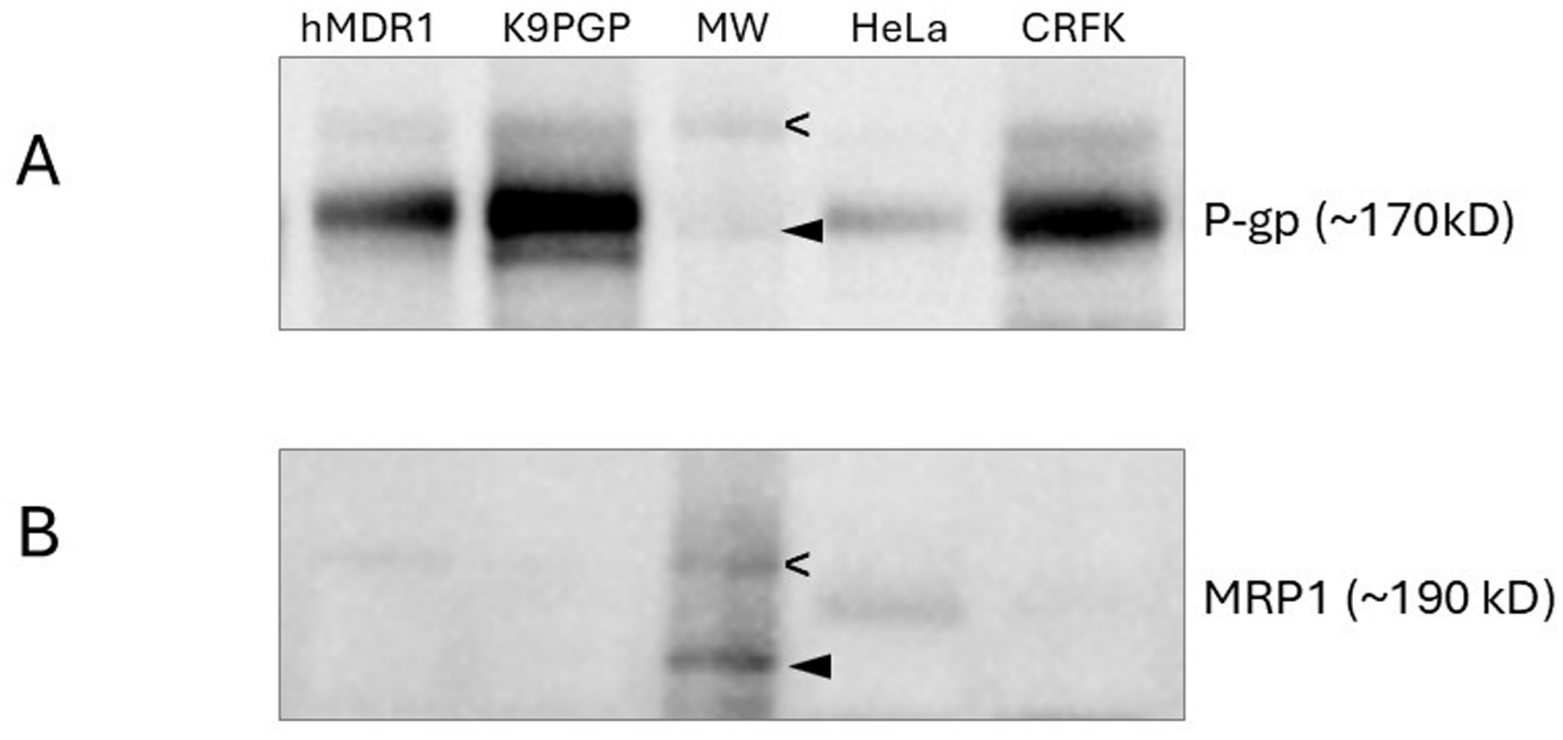
Western blots assessing P-gp **(A)** and MRP1 **(B)** expression in various cell lines. Molecular weight markers indicate 200 kD (open arrowhead) and 150 kD (closed arrowhead). hMDR1 MDCK cells (13), over- express human P-gp; K9PGP cells overexpress canine P-gp; HeLa cells are positive control cell line for MRP1 expression; CRFK cells are derived from feline renal cortex epithelium.

A western blot shows that the positive controls (hMDR1MDCK cells in which canine MDR1 has been knocked out and human MDR1 knocked in and MDCK PGP cells in which overexpression of canine P-gp was induced) and CRFK cells exhibit strong bands indicating robust P-gp expression (Figure 1A) while HeLa cells exhibit a weak band. The high level of P-gp expression in CRFK cells strongly suggest this cell line is an appropriate tool for interrogating drugs as feline P-gp substrates. In Figure 1B, MRP expression was confirmed only in HeLa cells, which have previously been shown to express MRP1^4^ (16), but not in CRFK cells.

### 3.2 Confirmation of functional P-gp in CRKF cells using rhodamine 123 efflux competition assay

**Figure 2:**
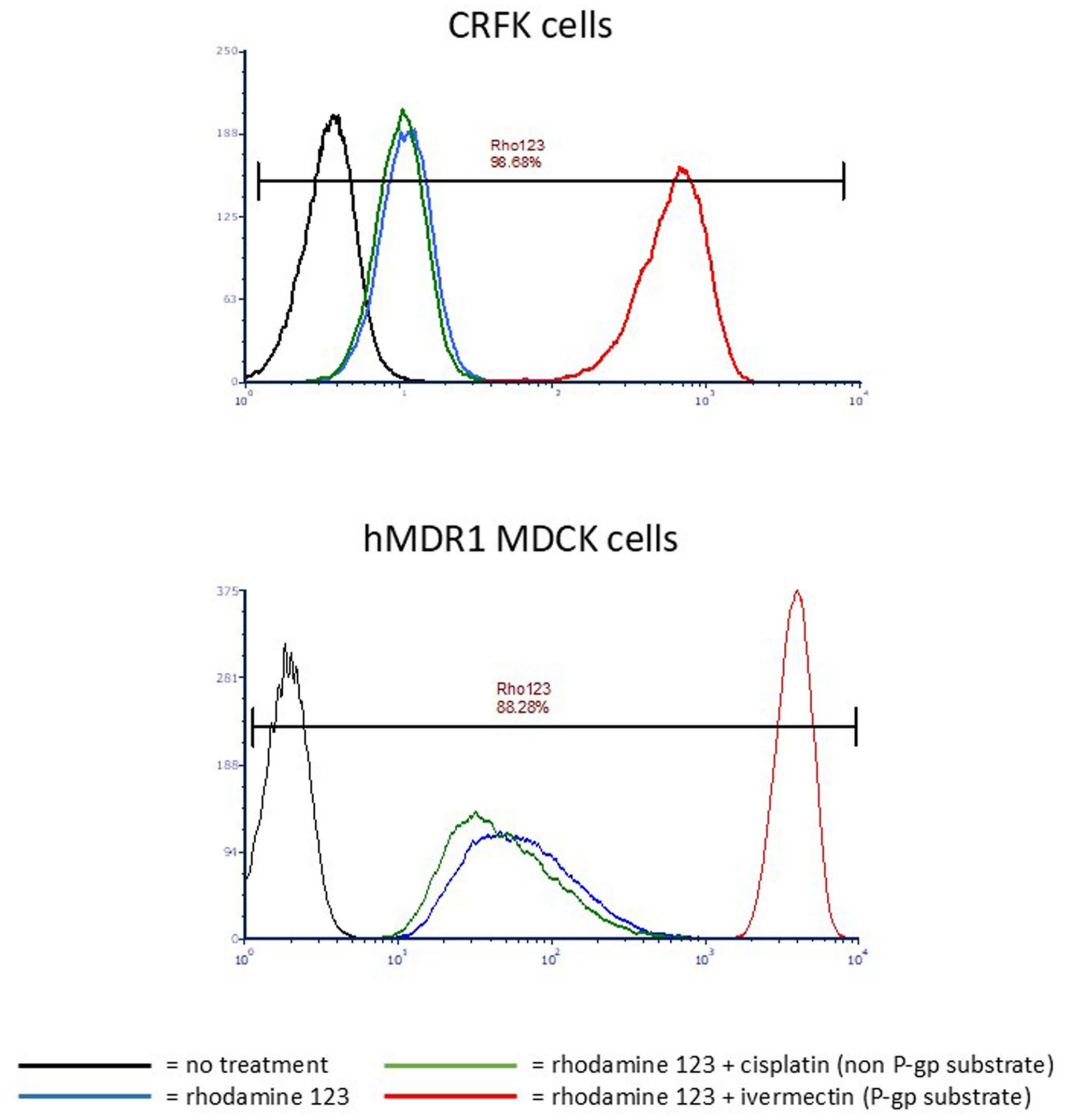
Histograms of CRFK or hMDR1 MDCK cells. X-axis = Log fluorescence intensity; Y-axis = cell count. Each peak represents a cell population that underwent a different treatment with the color corresponding to the treatment indicated in the key. Cells that highly express P-gp exhibit low mean fluorescence intensity (MFI) when treated with rhodamine123 alone because P-gp efficiently effluxes the compound, but high MFI when a P-gp substrate is co-incubated with rhodamine 123 because it competes with rhodamine 123 for P-gp-mediated efflux. MFI of untreated cells is < 10 (black); MFI of cells treated with rhodamine 123 only is ~10 for CRFKs and ~40 for hMDR1 (blue); MFI of cells treated with rhodamine123 and the non-P-gp-substrate cisplatin is ~10 for CRFKs and ~30 for hMDR1 MDCK (green); MFI of cells treated with rhodamine 123 and the P-gp substrate ivermectin is ~800 for CRFK and ~ 4,000 for hMDR1 MDCK (red).

P-gp function in CRFK cells was compared to that of the well-established human P-gp expressing cell line hMDR1 MDCK. Rhodamine 123 efflux was efficiently accomplished by both CRFK cells and hMDR1 MDCK cells when treated with rhodamine 123 alone or rhodamine 123 and cisplatin (non-P-gp substrate) (17) as indicated by low intracellular fluorescence intensity (Figure 2). Conversely, when the known P-gp substrate ivermectin was co-incubated with rhodamine 123, competition for P-gp-mediated efflux resulted in higher intracellular rhodamine 123 concentrations, as indicated by higher fluorescence intensity (Figure 2). These data demonstrate that P-gp expressed by CRFK cells is functional.

### 3.3 Flow cytometric assessment of experimental drugs as P-gp substrates

Table 1 lists the drugs for which feline P-gp substrate status was determined and the rationale for their inclusion, while Table 2 lists the concentrations tested. The range of experimental drug concentrations were intended to include plasma drug concentrations reported on drug labels or pharmacokinetic studies in cats or dogs if feline data was unavailable. When this information was unavailable, as for vinblastine and vincristine, concentrations were based on similar efflux studies in other cell lines (18). Ivermectin was used as the positive P-gp substrate control and cisplatin was used as the negative (non-P-gp substrate) control.

**Table 2:** Experimental drugs assessed as feline P-gp substrate, the resulting mean fluorescence intensity (MFI) ratio using either rhodamine 123 (RHO) or calcein AM (CAM), and the subjective interpretation.

Drug	MFI ratio (STD dev)		Interpretation	Concentration range (μM)
	Rhodamine 123	Calcein AM		
Amlodipine	1 (0.1)	4 (1.2)	Weak P-gp substrate	0.025, 0.25, **2.5**
Capromorelin	1 (0.1)	2 (0.1)	Weak P-gp substrate	0.05, 0.5, **5**
Cefovecin	1 (0.2)	1 (< 0.1)	Non P-gp substrate	30, 300, **3,000**
Cisapride	6 (0.8)	NA	Moderate P-gp substrate	0.1, 1, **10**
Cisplatin (−control)	1 (0.1)	1 (< 0.1)	Non P-gp substrate	**10**
Cyclosporine	44 (8.3)	NA	Strong P-gp substrate	0.01, 0.1, **1**
Doxycycline	1 (0.1)	1 (0.4)	Non P-gp substrate	1, 10, **100**
Emodepside	17 (5.1)	NA	Strong P-gp substrate	0.1, **1**, 10
Eprinomectin	13 (1.2)	15 (0.6)	Strong P-gp substrate	0.0033, 0.033, **0.33**
Ivermectin (+control)	59 (7.5)	NA	Strong P-gp substrate	0.001, 0.01, **0.1**
Loperamide (+control)	36 (8.2)	17 (0.6)	Strong P-gp substrate	**10**
Methylprednisolone	1 (0.1)	8 (1.2)	Moderate P-gp substrate	25, 50, **100**
Ronidazole	1 (0.1)	1 (< 0.1)	Non P-gp substrate	1.5, 15, **150**
Selamectin	46 (6.4)	NA	Strong P-gp substrate	0.8, **8**, 80
Vinblastine	25 (1.9)	NA	Strong P-gp substrate	1, 5, **10**
Vincristine	35 (2.9)	NA	Strong P-gp substrate	12.5, 25, **50**

MFI Ratio = (mean fluorescence intensity of experimental drug + fluorescent P-gp substrate)/mean fluorescence intensity of experimental drug alone at drug concentration indicated in bold; NA = not assessed. STD dev = standard deviation.

Because the P-gp substrates rhodamine 123 (R-site probe) (19) and calcein AM (P-site probe) (20) are intrinsically fluorescent, high intracellular concentrations of either fluorescent probe correspond to high mean fluorescence intensity (MFI). Cells that express high levels of P-gp such as CRFK cells will not have a high MFI after incubation with rhodamine 123 or calcein AM because P-gp efficiently effluxes these fluorescent P-gp substrates resulting in low MFI as seen in Figure 2. Consequently, If one calculates the ratio of the MFI of CRFK cells incubated with one of these fluorescent P-gp “probes” plus an experimental drug to the MFI of those cells incubated with the fluorescent P-gp probe alone [MFI (experimental drug + probe): MFI (probe alone)], a ratio of one is achieved when the experimental drug is not a P-gp substrate. Conversely, a high MFI ratio (> 10) is achieved when the experimental drug is a strong P-gp substrate that competes with rhodamine 123 at the R-site or calcein AM at the P-site of P-gp’s binding pocket (Table 2).

### 3.4 Assessment of calcein AM competitors as MRP substrates

Because calcein AM may be an MRP1 substrate, experimental drugs (amlodipine and methylprednisolone) that competed with calcein AM were assessed as MRP1 substrates in a competitive efflux assay using the fluorescent MRP1 substrate CFDA and selective MRP1 inhibitor MK571 as the positive control (14). The MFI ratios for MK571, amlodipine, and methylprednisolone were 28.3, 1.0 and 1.1, respectively. These MFI ratios indicate that neither amlodipine nor methylprednisolone are MRP1 substrates. The MFI ratio for MK571 in HeLa cells, which showed higher MRP1 expression than CRFK cells on the western blot, was nearly 4-fold greater than that in CRFK cells.

## 4 Discussion

Knowing a drug’s P-gp substrate status is critically important for feline drug safety. The feline MDR1 mutation (ABCB11930_1941del TC) has been documented in the US and Europe at a frequency of up to 6% in Maine Coon cats (Anderson *et al*, 2022) and roughly 1% in non-pedigreed cats (11, 21), with one study identifying ABCB11930_1941del TC in 4% of non-pedigreed cats (7). Knowing a drug’s P-gp substrate status may be equally important for drug safety even for cats without the feline MDR1 mutation, the remaining ~95% of the feline population, because of acquired P-gp deficiency. Acquired P-gp deficiency can occur when two P-gp substrate drugs are concurrently administered, creating competition for P-gp-mediated efflux at the blood brain barrier and biliary canaliculi (2).

Expression and function of P-gp endogenously expressed by CRFK cells were confirmed by western blot and competitive efflux studies, respectively. Based on the western blot band intensity, endogenous P-gp expression in CRFK cells appears to be slightly greater than in hMDR1 P-gp cells, the Madin-Darby canine kidney cell line transfected with human P-gp and slightly less than MDCK PGP cells, the Madin-Darby canine kidney cells induced to overexpress canine P-gp. The CRFK epithelial cell line was derived from the renal cortex of a female cat so likely consists of renal tubular cells which have been documented to have robust P-gp expression (3). Because MRP1 and P-gp can share substrates it was important to rule out potential MRP1-mediated efflux activity. CRFK cells appear to express minimal MRP based on western blot results. While CRKF cells did demonstrate some MRP1-mediated efflux activity, it was substantially less than that of HeLa cells.

Rhodamine 123 is an intrinsically fluorescent P-gp substrate that interacts with a site called the R-site within P-gp’s binding pocket, so-named because it binds Rhodamine (22). When cells expressing functional P-gp are incubated with rhodamine 123, its intracellular concentration is low due to P-gp-mediated efflux, and this is reflected in a relatively low fluorescence intensity reading when cells are subjected to flow cytometry. However, if another R-site P-gp substrate is co-incubated with rhodamine 123, the resulting competition for P-gp-mediated efflux results in higher intracellular concentrations of rhodamine 123, generating a high fluorescence intensity when cells are subjected to flow cytometry. Figure 1, which shows flow cytometry histograms for CRFK and hMDR1 PGP cells, illustrates P-gp’s effect on rhodamine 123 alone (relatively low fluorescence intensity) and rhodamine 123 co-incubated with the positive control P-gp substrate ivermectin (high fluorescence intensity). Cisplatin, a drug that is not a P-gp substrate thereby serving as a negative control, has no effect on fluorescence intensity. The resulting MFI ratio of ivermectin in CRFK cells was 59 (indicating that ivermectin is a strong substrate for feline P-gp) while the MFI for cisplatin was 1, confirming that cisplatin is not a feline P-gp substrate.

Experimental drugs were selected based on their use in cats, reported sporadic neurological adverse effects in cats, potential for the drug to have caused a P-gp-mediated adverse drug reaction in a cat, or because the drug is a P-gp substrate in other species (Table 1). Many of these drugs competed strongly with rhodamine 123 efflux, as indicated by a MFI ratio greater than 10, including cyclosporine, emodepside, eprinomectin, loperamide, selamectin, vinblastine, and vincristine. Each of these strong feline P-gp substrates has been shown to be a P-gp substrate in other species so results are not surprising. Cisapride, with a MFI ratio of 6, is considered a moderate P-gp substrate at the R-site. Interestingly, there are reports of sporadic neurological toxicosis in cats treated with cisapride^5^ and neurological effects observed in cases of overdose (23). Several drugs, including amlodipine, capromorelin, cefovecin, doxycycline, methylprednisolone and ronidazole, did not compete with rhodamine 123 for P-gp-mediated efflux (MFI ratios of 1), so were determined not to be R-site P-gp substrates.^6^

For drugs that were not R-site P-gp substrates, the assay was repeated using the P-site fluorescent P-gp substrate calcein AM. As indicated in Table 2, methylprednisolone generated a MFI ratio of 8 so was designated a moderate feline P-gp substrate. Methylprednisolone appears to have contributed to a drug interaction involving eprinomectin, a strong substrate of the feline P-gp P-site (10). Amlodipine (MFI ratio 4) and capromorelin (MFI ratio 1.5, rounded to 2) were designated weak P-gp substrates, while cefovecin and ronidazole (MFI ratios of 1) were determined to not be feline P-gp substrates.

Because calcein AM is both a P-gp P-site substrate and an MRP1 substrate, it was important to determine if the experimental drugs that competed with calcein AM were MRP1 substrates. Amlodipine and methylprednisolone competed with calcein AM so they were assessed as MRP1 substrates using the selective, fluorescent MRP1 substrate CFDA. Generating MFI ratios of 1.0 and 1.1, respectively, neither amlodipine nor methylprednisolone are feline MRP1 substrates indicating that the efflux ratios generated using calcein AM can be attributed solely to P-gp.

The designations of strong (MFI ratio ≥ 10), moderate (5 < MFI ratio < 10), and weak (2 < MFI ratio < 5) P-gp substrate status may be somewhat arbitrary with respect to the cut-off values. However, P-gp substrates with higher MFI ratios (i.e., macrocyclic lactones, vinca alkaloids, emodepside, cyclosporine) as determined by our efflux assay tend to be associated with drugs that are well-documented to cause serious adverse effects in animals with P-gp deficiency. It is the authors’ collective opinion that these designations are useful from a clinical perspective when weighing the risks and benefits of treating a P-gp deficient patient with a P-gp substrate and for determining appropriate dose reductions of P-gp substrates in P-gp deficient patients.

Two previous studies have examined feline P-gp efflux via flow cytometry. A 2015 paper (24) describes a feline lymphoma cell line induced to overexpress P-gp using doxorubicin. Rhodamine 123 was used in a similar efflux competition assay. Two drugs in that study, cyclosporine and ivermectin, overlapped with our study results between those studies were consistent (both competed with rhodamine 123). One other study (9) assessed feline P-gp function using a direct, non-competitive assay. In this study, HEK cells were transfected with the feline MDR1 gene. Direct comparisons to our studies cannot be made because there were no overlapping drugs in the two studies. That study used digoxin, quinidine, taninolol, fexofenadine and paclitaxel, drugs considered to be classic human P-gp substrates but certainly less clinically relevant for cats than the drugs used in our study. Nonetheless, feline P-gp transported those drugs. It would be interesting to compare results using the direct assay (9) to a competitive efflux assay. While the direct assay does not allow assessment of specific binding sites, it may offer other advantages. For example, if a drug has a lower affinity for P-gp than the fluorescent probe, and is tested at lower concentrations than the fluorescent probe, the competitive efflux assay may not identify it as a P-gp substrate. This shortcoming can be overcome by ensuring that adequate concentrations of test drugs are assessed in competitive efflux assays.

Several potential limitations of this study should be mentioned. First, media used in these experiments contained 10% fetal bovine serum. It is possible that results may be varied if experiments had been conducted using serum-free media. However, these possible variations may have been minimized because an MFI ratio was used as the determining factor of P-gp substrate status whereby variation is minimized by its contribution to both the numerator and denominator. Another potential limitation is the possibility of rhodamine 123 being a substrate for OCT1 and/or OCT2 as reported in other species. Even if rhodamine 123 is a substrate for these transporters, the impact on results would likely be minimal for several reasons. First, both OCT1 and 2 transport bi-directionally. While net influx seems to predominate, the magnitude of substrate influx (2-fold) relative to P-glycoprotein-mediated efflux (often exceeding 10-fold) would blunt their potential impact on results. Second, cisplatin, a negative control for P-gp, has been reported to be a substrate for OCT2. There was no difference in the intracellular rhodamine 123 concentration when CRFK cells were incubated with rhodamine 123 alone or co-incubated with cisplatin, ruling out any potential contribution of OCT2. Third, because the study design incorporates a ratio in which potential contributions of OCT1 and OCT2 are incorporated into both the numerator and denominator, these potential contributions are minimized. Importantly, results reported here have accurately categorized the drugs for which there is *in vivo* evidence of feline P-gp substrate status (i.e., ivermectin, eprinomectin), presumed P-gp substrates based on canine and human data (selamectin, loperamide, vincristine, vinblastine) as well as the negative control (cisplatin) so it seems that there is “wiggle room” built into the system.

## 5 Conclusion

Experiments described herein yielded important results. Specifically, the documentation of a cell line expressing feline P-gp that can be used to determine if drugs are substrates for feline P-gp and application of that cell line to identify several clinically important drugs as feline P-gp substrates. The availability of this cell line should enable screening of drugs intended for use in cats to determine their P-gp status as is customary for new human drugs (25). This knowledge can be used to prevent adverse drug reactions in cats with intrinsic or acquired P-gp deficiency such as those that have occurred with eprinomectin-containing products (10, 11). Additionally, since species differences in P-gp substrates are known to exist, it is inappropriate to assume that drugs identified as human P-gp substrates would necessarily be feline P-gp substrates.

## Data Availability

The raw data supporting the conclusions of this article will be made available by the authors, without undue reservation.

## Footnotes

^1^Broadline

^2^Centraguard

^3^NexGard Combo

^4^https://www.ptglab.com/products/MRP1-Antibody-27825-1-AP.htm#:~:text=37%C2%B0C%20incubated%20HeLa%20cells%20were%20subjected%20to%20SDS,in%20each%20testing%20system%20to%20obtain%20optimal%20results (accessed May 23, 2025)

^5^https://cats.com/cisapride-for-cats (accessed April 23, 2024)

^6^https://docs.boehringer-ingelheim.com/NexGard%C2%AE%20COMBO%20%28esafoxolaner%2C%20eprinomectin%2C%20and%20praziquantel%20topical%20solution%29.pdf (accessed April 18, 2024)
