# Correction: Assessment of clinically relevant drugs as feline P-glycoprotein substrates

**DOI:** 10.3389/fvets.2026.1867072

**Published:** 2026-05-22

**Authors:** Katrina L. Mealey, Neal S. Burke

**Affiliations:** Program in Individualized Medicine (PrIMe), Department of Veterinary Clinical Sciences, Washington State University, Pullman, WA, United States

**Keywords:** P-glycoprotein, MDR1, eprinomectin, cisapride, feline

In the published article, there was an error in Table 2 as published. The header of the last column, Concentration Range, features an incorrect unit abbreviation. Instead of MM, it should be μM. The corrected Table 2 and its caption appear below.

**Table 2:** Experimental drugs assessed as feline P-gp substrate, the resulting mean fluorescence intensity (MFI) ratio using either rhodamine 123 (RHO) or calcein AM (CAM), and the subjective interpretation.

Drug	MFI ratio (STD dev)		Interpretation	Concentration range (μM)
	Rhodamine 123	Calcein AM		
Amlodipine	1 (0.1)	4 (1.2)	Weak P-gp substrate	0.025, 0.25, **2.5**
Capromorelin	1 (0.1)	2 (0.1)	Weak P-gp substrate	0.05, 0.5, **5**
Cefovecin	1 (0.2)	1 (< 0.1)	Non P-gp substrate	30, 300, **3,000**
Cisapride	6 (0.8)	NA	Moderate P-gp substrate	0.1, 1, **10**
Cisplatin (–control)	1 (0.1)	1 (< 0.1)	Non P-gp substrate	**10**
Cyclosporine	44 (8.3)	NA	Strong P-gp substrate	0.01, 0.1, **1**
Doxycycline	1 (0.1)	1 (0.4)	Non P-gp substrate	1, 10, **100**
Emodepside	17 (5.1)	NA	Strong P-gp substrate	0.1, **1**, 10
Eprinomectin	13 (1.2)	15 (0.6)	Strong P-gp substrate	0.0033, 0.033, **0.33**
Ivermectin (+control)	59 (7.5)	NA	Strong P-gp substrate	0.001, 0.01, **0.1**
Loperamide (+control)	36 (8.2)	17 (0.6)	Strong P-gp substrate	**10**
Methylprednisolone	1 (0.1)	8 (1.2)	Moderate P-gp substrate	25, 50, **100**
Ronidazole	1 (0.1)	1 (< 0.1)	Non P-gp substrate	1.5, 15, **150**
Selamectin	46 (6.4)	NA	Strong P-gp substrate	0.8, **8**, 80
Vinblastine	25 (1.9)	NA	Strong P-gp substrate	1, 5, **10**
Vincristine	35 (2.9)	NA	Strong P-gp substrate	12.5, 25, **50**

MFI ratio, (mean fluorescence intensity of experimental drug+fluorescent P-gp substrate)/mean fluorescence intensity of experimental drug alone at drug concentration indicated in bold; NA, not assessed; STD dev, standard deviation.

The original version of this article has been updated.

